# Climate and permafrost effects on the chemistry and ecosystems of High Arctic Lakes

**DOI:** 10.1038/s41598-017-13658-9

**Published:** 2017-10-16

**Authors:** K. E. Roberts, S. F. Lamoureux, T. K. Kyser, D. C. G. Muir, M. J. Lafrenière, D. Iqaluk, A. J. Pieńkowski, A. Normandeau

**Affiliations:** 1 Department of Geography and Planning, Queen’s University, Kingston, ON K7L 3N6 Canada; 2 Department of Geological Sciences and Geological Engineering, Queen’s University, Kingston, ON K7L 3N6 Canada; 3 Environment and Climate Change Canada, Aquatic Contaminants Research Division, Burlington, ON Canada; 4 Resolute Bay, NU, Canada; 5 Department of Physical Sciences, MacEwan University, Edmonton, AB T5J 4S2 Canada; 6 School of Ocean Sciences, College of Natural Sciences, Bangor University, Anglesey, LL59 5AB UK; 7 Natural Resources Canada, Geological Survey of Canada Atlantic, Dartmouth, NS B2Y 4A2 Canada

## Abstract

Permafrost exerts an important control over hydrological processes in Arctic landscapes and lakes. Recent warming and summer precipitation has the potential to alter water availability and quality in this environment through thermal perturbation of near surface permafrost and increased mobility of previously frozen solutes to Arctic freshwaters. We present a unique thirteen-year record (2003–16) of the physiochemical properties of two High Arctic lakes and show that the concentration of major ions, especially SO_4_^2−^, has rapidly increased up to 500% since 2008. This hydrochemical change has occurred synchronously in both lakes and ionic ratio changes in the lakes indicate that the source for the SO_4_^2−^ is compositionally similar to terrestrial sources arising from permafrost thaw. Record summer temperatures during this period (2003–16) following over 100 years of warming and summer precipitation in this polar desert environment provide likely mechanisms for this rapid chemical change. An abrupt limnological change is also reflected in the otolith chemistry and improved relative condition of resident Arctic char (*Salvelinus alpinus*) and increased diatom diversity point to a positive ecosystem response during the same period.

## Introduction

The aquatic ecosystems of High Arctic freshwater lakes are strongly influenced by the presence of persistent ice cover and permafrost. These factors cause cold lakes to be sensitive to small changes in climatic conditions^1^. Many studies indicate that Arctic regions are undergoing rapid climatic and permafrost change^2–6^. Small aquatic systems such as ponds have demonstrated abrupt physiochemical and ecosystem responses^7^, while larger lakes are thought to be more likely to gradually respond due to their larger volume^8^. Climate models consistently project not only warming temperatures in the Arctic, but also increased precipitation that can alter hydrological regimes by shifting runoff contribution from early season snowmelt to later season rainfall events^9^. Climate warming has the potential to impact the hydrological regimes of lakes directly by thawing the surrounding permafrost, altering subsurface water exchanges, and in some cases causing lake drainage^6^ as well as through permafrost degradation and thermokarst in the watershed^10^. Modelling results indicate that a significant increase in near surface permafrost thaw and thermokarst is expected to continue across the Arctic^11^ and will have significant effects on the chemical composition of freshwater lakes by allowing previously immobile soluble ions in near surface permafrost that is seasonally thawed to enter rivers and lakes^12,13^, particularly because the deepest seasonal thaw is often both ice and solute-rich^14^. Recent studies have documented near surface permafrost degradation leading to altered shallow hydrological pathways^6^, increased groundwater contribution and solute and nutrient delivery to Arctic river basins^15,16^, but the impact has not been demonstrated in relatively large lake systems mostly due to their greater volume and slow water turnover. Moreover, the effects of permafrost thaw are mostly commonly documented in regions with relatively warm permafrost (> −5 °C), but are increasingly observed in areas with colder permafrost (<−10 °C)^5,6^.

In addition to physiochemical changes, significant changes to aquatic biota have been observed^7^ and are predicted^17^. Diatom diversity and populations are used as indicators of environmental change as each species has specific ecological preferences related to physiochemical conditions^18,19^. With continued climatic warming, a reduction in ice cover and duration may lead to community shifts of benthic species to planktonic species^19,20^. In addition to changes to primary producers, Arctic char represent the top predator in many Arctic lakes. Warming water temperatures may increase primary productivity, which in turn may lead to increased char body weight and length^17^. Yet, there are few studies that demonstrate how rapid limnological change may impact fish species.

**Figure 1:**
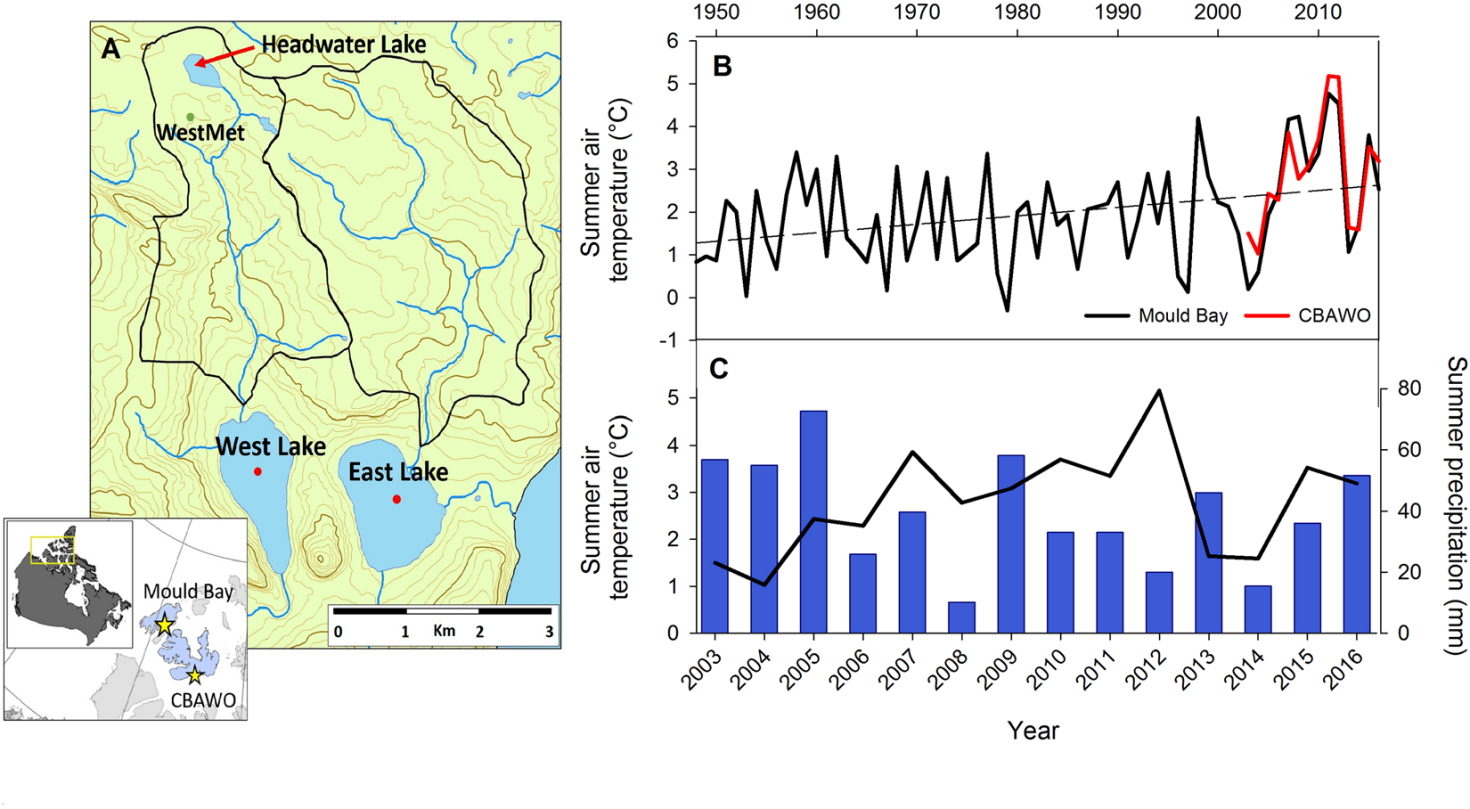
(**A**) Regional Map of Cape Bounty, Melville Island, NU and Mould Bay, Prince Patrick Island, NT. (**B**) Regional summer (June, July, August) temperatures since 1948 (Mould Bay), and (**C**) summer air temperatures and precipitation at CBAWO 2003–15 (WestMet). Map prepared with data from http://www.nrcan.gc.ca/earth-sciences/geography/atlas-canada using ArcGIS v.10.4, and contains information licensed under the Open Government Licence – Canada.

This study documents recent rapid chemical change in two similar adjacent High Arctic lakes (Fig. 1) using a long-term data set from the Cape Bounty Arctic Watershed Observatory (CBAWO*)*, a limnological and hydrological research site in the Canadian Arctic Archipelago *(74°50’N, 109°30’W*). The physical and chemical properties of both the unofficially named East and West Lakes have been monitored from 2003 to 2016 and therefore, this study represents the longest seasonal limnological and hydrochemical record in the High Arctic. To further assess the impact of these environmental changes on the ecology of the lakes, the temporal changes in the chemistry of Arctic char otoliths (ear bones) were compared to the temporal changes in lake hydrochemistry. In addition, lake diatom communities were enumerated in 2004^21^ and 2014 to determine changes in species composition during this period of rapid change.

## Site Description

The adjacent East and West Lakes in this study have 11.6 km^2^ and 8.0 km^2^ watersheds, respectively, with prostrate shrub tundra vegetation and a total relief of 100 m. Permafrost in this High Arctic setting is cold (−14 °C), continuous^22^ and likely exceeds 500 m in thickness^23^, and the active layer reaches a depth of approximately 50–70 cm in the summer season. The East (1.6 km^2^) and West (1.4 km^2^) Lakes have maximum depths of 31 m and 34 m, similar volumes of 0.02 km^3^ and receive approximately 2.6–6.0% replacement^21^ from inflowing rivers annually (Fig. 1).

The climate of the region is amongst the coldest in North America and classified as a High Arctic polar desert. Mean monthly temperatures only rise above freezing in the months of June (0.2 °C), July (4.0 °C), and August (0.9 °C) as measured by the nearest long term monitoring station Mould Bay, NWT. The region is also dry, receiving approximately 160 mm or less of precipitation annually. Record July air temperatures in 2007 at CBAWO resulted in deep surface thaw of soils (~1 m) and in combination with rainfall that generated over 100 slope failures (active later detachments, ALD) that cover 2.8 and 1.0% of the West and East catchments, respectively^24^. Runoff monitoring from disturbed tributaries of the West River demonstrated enhanced solute and suspended sediment fluxes^9^ although initial downstream effects in the lakes were limited^25^. Since 2007, regional warming has resulted in record warm air temperatures and when compared with the nearest long-term monitoring station (Mould Bay 300 km northwest 1948–2016), a clear increase in mean summer air temperatures of approximately 2 °C is evident in the region and four of the warmest summers on record have occurred in the past decade (Fig. 1). Associated measurements of hydrological inflows have also been undertaken but autumn (August) discharge data is unavailable^9^.

## Results and Discussion

Both lakes are monomictic and subject to ice cover until late July or early August. The most substantial physical change recorded by the lakes is an abrupt rise in turbidity in West Lake from ~4 Nephelometric Turbidity Units (NTU) in 2006, to over 250 NTU in 2015 (Supplementary Fig. S1), while the East Lake turbidity has consistently remained <6 NTU for the entire study period. The increase in turbidity in West Lake is due to three subaqueous slumps that occurred in September 2008, December 2011, and February 2012, unrelated to river inflows or terrestrial permafrost disturbance^25^.

**Figure 2:**
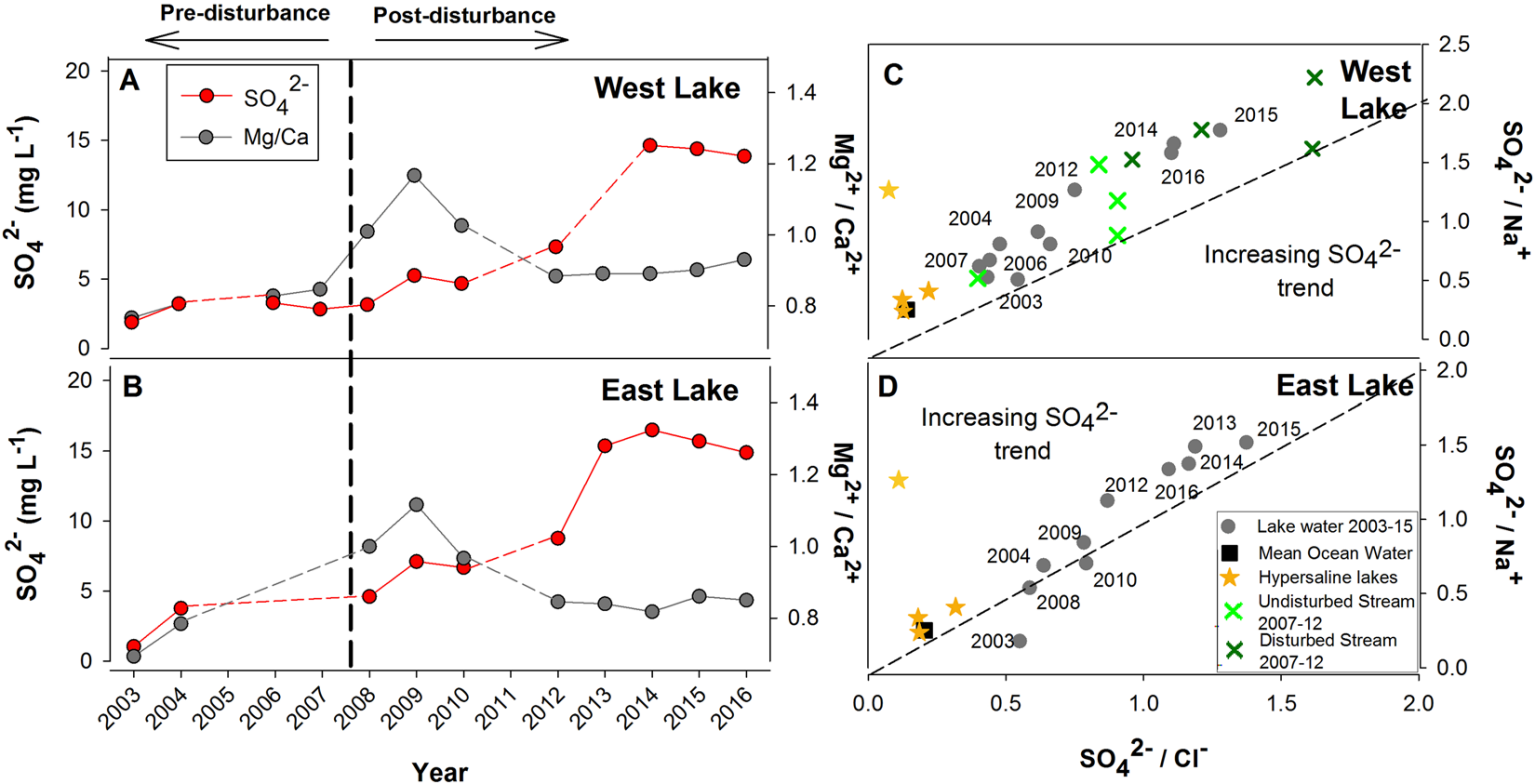
(**A**) Concentrations of SO_4_^2−^ (mg/L) and Mg^2+^/Ca^2+^ ratios (mg/L) over time for (**A**) West and (**B**) East. SO_4_^2−^/Na^+^ and SO_4_^2−^/Cl^−^ ratios of (**C**) West and (**D**) East Lakes compared to marine water, regional hypersaline lakes and West catchment streams (disturbed = Ptarmigan, Undisturbed = Goose, 2007–09, Lamoureux and Lafrenière (2013), 2012 Unpublished Data). Dashed line indicates a 1:1 ratio.

Water column hydrochemistry in both lakes was generally uniform with depth (with slight increases in the bottom 1–5 m) and characterized by a gradual overall increase in specific electrical conductivity from 38 to102 µS/cm in West Lake and from 29 to 136 µS/cm in East Lake from 2004 to 2016 (Supplementary Fig. S2). In particular, SO_4_^2−^ concentrations increased from approximately 3 to 15 mg L^−1^ ( + 500%) in West Lake during 2006 to 2016 and from 5 to 17 mg L^−1^ ( + 340%) in East Lake from 2008 to 2016 (Fig. 2). One-way ANOVA indicates that the change between the pre-disturbance concentrations (2003–2006) and post-disturbance concentrations (2012–2015) were found to be statistically significant for the East and West Lakes (F = 17.343, df 5, p = 0.014 and F = 14.576, df 5, p = 0.019, respectively). The SO_4_^2−^ concentration change was not gradual, with the largest increases occurring in 2009 and particularly 2013. The relationship between SO_4_^2−^:Cl^−^ and SO_4_^2−^:Na^+^ indicates that SO_4_^2−^ is increasing largely independently of the other major ions. By contrast, other major ions show gradual increases in concentration over this period (Supplementary Fig. S3). This pattern is also demonstrated by the other major ions such as a short-lived increase in lake water Mg^2+^/Ca^2+^ (Fig. 2). A similar pattern followed the impact of the upstream 2007 permafrost disturbance episode and is consistent with previously-reported surface water runoff from disturbed catchments^13^. Conversely, the large increases in the SO_4_^2−^ concentrations in the lakes following 2009 are not directly associated with 2007 catchment permafrost disturbance. The prominent increase in SO_4_^2−^ is accompanied by moderate increases in Cl^−^, although there is not a matching major cation to balance this increase. In both lakes, there have been decreases in total Ba, Fe, Mn, and Zn and increases in Ca, Mg, K, Na, and Sr between 2003 and 2015 (Supplementary Fig. S4). Aluminum is the only element that shows an inconsistent response between lakes, with an increase in Al in the West Lake most likely due to the increase in mineral particulates associated with sustained high turbidity (Supplementary Figs S1 and S4).

The changes in the chemical compositions of the lakes at CBAWO are directed away from the composition of mean ocean water and coastal hypersaline lakes in the region (Fig. 2) and strongly suggest that the source of the solutes is not marine^26,27^. Rapid chemical alteration related to evaporative enrichment of solutes during climate warming has been documented for small Arctic ponds^7,8,28^, but not for larger lake systems. Given that the SO_4_^2−^ waters at CBAWO are compositionally similar to the disturbed tributary waters^13^, the source is likely increased solute mobilization and drainage from both the deep active layer and the upper permafrost driven by increased summer temperatures and precipitation. Sustained deep seasonal thaw likely provides the mechanism for increased solute-rich soil water inflows to both lakes. Soil cores (obtained pre-thaw 2012) indicate a substantial increase in ion concentrations (especially SO_4_^2−^, Cl^−^, and Ca^2+^) occurs at 80–100 cm depth, below the typical maximum active layer depth of 50–70 cm^29^. For example, SO_4_^2−^ increases from c. 3–5 mg/100 g (~22 Mg/km^2^) soil in the active layer to 8–11 mg/100 g (~54 Mg/km^2^) soil immediately below the active layer. Each major increase in lake SO_4_^2−^ load occurs in the year following elevated summer air temperatures (2007, 2011, and 2012) and in particular, years with substantial late-season rainfall events such as 2012^29^. These years represent both the warmest summers since records began in the region in 1949, and likely also for several millennia across the region^3^. The largest SO_4_^2−^ increase occurred between 2012 and 2013, following two consecutive warm summers in 2011 and 2012.

Degradation of near-surface permafrost would occur across the landscape and may also deliver solutes via subsurface flow^13^ and has the potential to release sufficient sulfur to rapidly alter the composition of the lakes. Moreover, active layer depth is highly sensitive to small shifts in annual climatic conditions and explains why SO_4_^2−^ concentrations have stabilized in the relatively cool years of 2013–16 (Fig. 1). On average, SO_4_^2−^ concentrations in the West and East Lakes increased by 1.4 and 2.1 mg L^−1^ each year, or approximately 30 and 43 Mg of SO_4_^2−^ added each year to the West and East Lakes, respectively from 2006–16. The ratio of SO_4_^2−^ flux (West:East) of 0.70 is similar to the catchment area ratio (0.69) and is consistent with a proportionate landscape-wide contribution to the lakes of 3.5–3.7 Mg km^−2^ SO_4_^2−^. Thus, thermally-driven near-surface permafrost thaw in the lake catchments has the potential to release ions from the permafrost. This thaw mechanism is consistent with enhanced solute flushing to the lakes and is expected to be widespread across the landscape compared to the highly localized surface disturbances^13^ and hence, holds the potential to deliver large amounts of solutes to the lakes.

The presence of permafrost typically restricts shallow lateral and groundwater inflows to the lakes and is especially true in regions of thick continuous permafrost, where most small and medium lakes cannot support through taliks (unfrozen ground that extends to the base of the permafrost) that are more common in discontinuous permafrost regions^30^. As permafrost in this High Arctic region is cold, thick (~500 m)^23^ and continuous, permafrost extent is unlikely to completely thaw during the next century^5,17^. Instead, evidence for near-surface permafrost degradation is widespread^12,24^ and permafrost warming has been observed in boreholes^31^. The input from surface and shallow subsurface flows can be enhanced through active layer deepening and increased summer precipitation. Climate models have predicted Arctic precipitation increases of 7.5–18.1% mostly in autumn and winter, and less so in summer^4^. However, increasingly warm summer temperatures may lead to late-summer/early-autumn precipitation falling as rain as opposed to snow, which may shift relative runoff importance from snowmelt to rainfall^9^. We note that rainfall runoff in the catchment is important because it provides widespread connectivity and activation of small tributaries. These summer rainfall events that occur later in the season can cause disproportionally large biogeochemical responses because the seasonal thaw is deepest at this time allowing for maximum flushing of solutes into downstream rivers and lake systems^9,29,32^.

**Figure 3:**
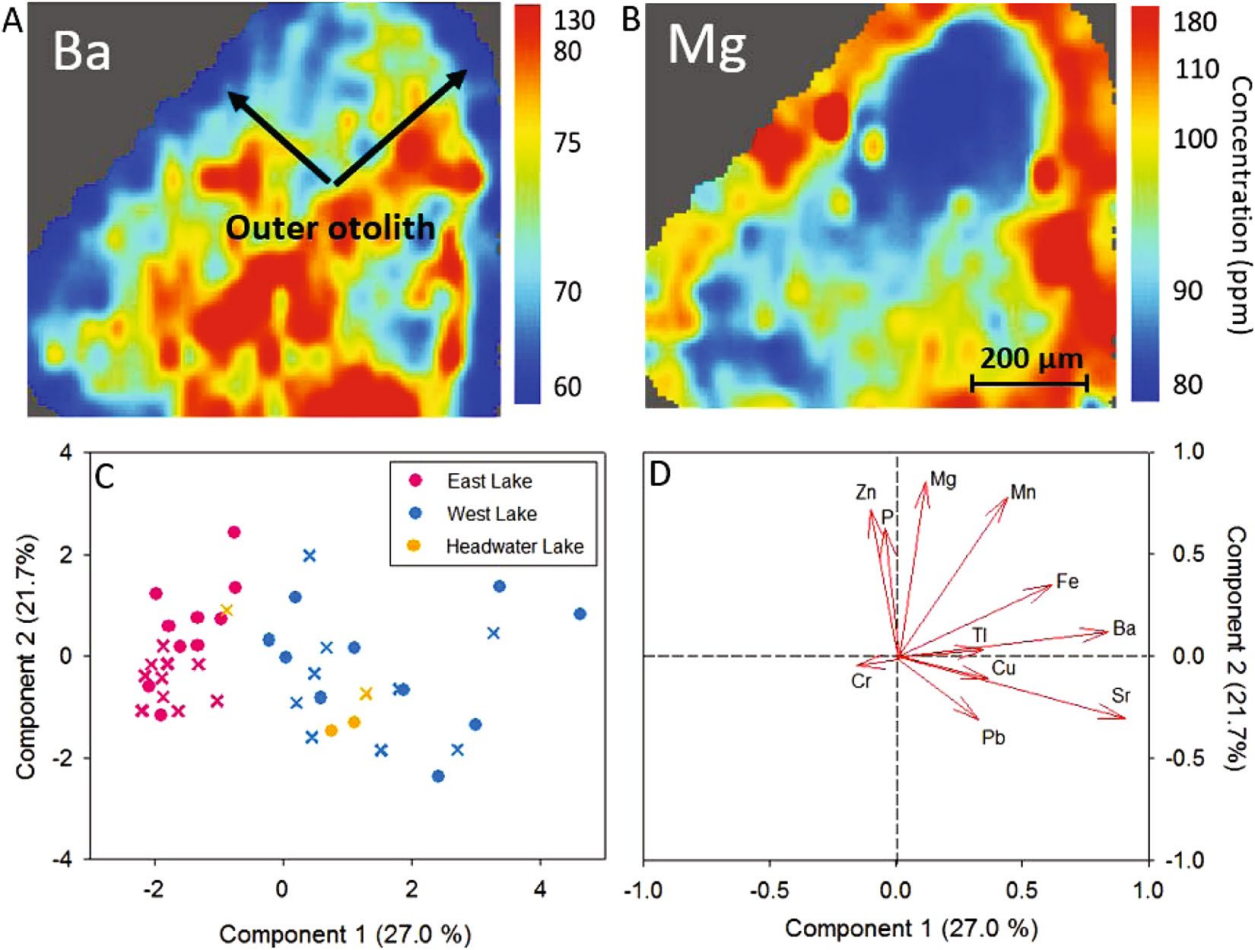
Elemental maps (**A**) Ba and (**B**) Mg of East Lake otolith sampled in 2015. (**C**,**D**) Principal component analysis of inner/outer otolith data 2013–2015: 10 fish from West, 10 fish from East, and 2 fish from Headwater. Circle = Inner Otolith, X = Outer Otolith.

To evaluate the ecosystem effect of increased solutes, elemental scans of Arctic char (*Salvelinus alpinus*) otoliths analysed from East Lake, West Lake, and Headwater Lake which is a small and shallow (4 m) tributary of West Lake upstream of thermokarst disturbance. Most elements including Sr, Cu, and Zn showed no consistent trends suggesting no temporal variation in uptake (Fig. 3). However, all otoliths from the East and Headwater Lakes record an abrupt decrease in Ba concentrations and a corresponding increase in Mg concentrations in the outer 100–200 µm of the otolith (Fig. 3). This geochemical pattern is less consistent in West Lake otoliths where only one of three fish show an increase in Mg around the outer rim and two showed a strong decrease in Ba. Although the width of this outer rim varies depending on the individual, it represents the last 5–8 years of life, which corresponds to the abrupt change in chemical composition of both lake systems and is consistent with the 80% increase in lake water Mg and 90% decrease in Ba concentrations in both lakes (Supplementary Fig. 4). These hydrochemical changes are also broadly indicated by analysis of inner (early life) and outer (late life) analysis of a larger set of 22 otoliths from all three lakes (10 from West, 10 from East, 2 from Headwater). Principal component analysis indicates that the most important elements driving variance along the positive axis of PC1 are Ba, Sr, and Fe, while PC2 is most strongly influenced by Mg, Zn, and P. Notably, char from the West and East lakes can be separated by the positive and negative axes of PC1 and the early and late life phase of the East Lake are separated by the positive and negative axes of PC2 (Fig. 3). Otoliths from Headwater Lake show a similar elemental response as the West Lake population.

**Figure 4:**
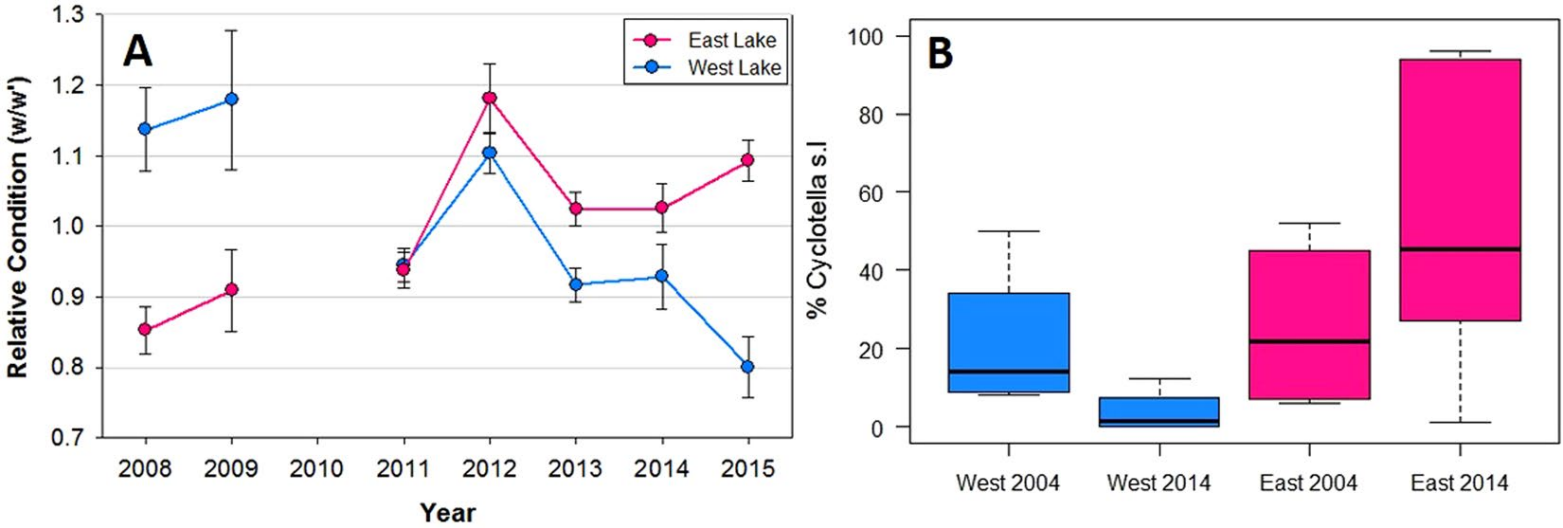
(**A**) Relative Fish Condition for East and West Lake 2008–15. Note: sample data are not available from 2010. (**B**) Percent planktonic shifts (*Cyclotella pseudostelligera* and *Cyclotella rossii, grouped as Cyclotella s.l*.) 2004 and 2014.

Although the lakes have shown similar chemical responses, the otolith compositions indicate that individual fish have been responding to limnological change differently in each lake. The declining relative condition (see methods) of fish from the West Lake is attributed to decreasing fish mass relative to length over time whereas fish masses in the East Lake are increasing over the same interval (Fig. 4). Given the similar hydrochemical changes in both lakes, we attribute the difference in fish condition to turbidity in the West Lake. Char are visual predators and rely on vision to locate prey^33^. With turbidity >100 times higher than the East Lake, visibility has been substantially limited since September 2008 when the first subaqueous slump occurred. In the East Lake, the fish are not inhibited by water column turbidity and the increase in health status likely reflects warmer water temperatures and reduced ice cover duration during this period. Perhaps more importantly, the increase in catchment soil flushing to the lakes has likely contributed to enhanced growth conditions for the fish through concomitant nutrient delivery^34^. Hence, the rapid physiochemical changes that have occurred in the lakes appear to have contributed to a sustained improvement in fish health in the East Lake, which is consistent with projected outcomes expected across the Arctic^17^.

Aquatic ecosystem changes are also reflected by diatoms, which show striking shifts between 2004^21^ and 2014 (Fig. 4). Whereas the 2004 littoral diatoms in both lakes and the 2014 West Lake assemblages are primarily benthics with a minor planktonic component (mostly *Cyclotella rossi*). By contrast, as much as 50% of the 2014 East Lake littoral assemblages are comprised of *Cyclotella pseudostelligera*, a small, fast-growing planktonic centric that may outcompete benthic species in changing environments due to more efficient nutrient and light uptake, low sinking velocities, and fast reproduction^19^. The East Lake diatom compositional shifts 2004–14 may thus be a result of changing vertical mixing, light, and nutrient regimes favorable to planktonic diatoms. Though diatom changes may be indirectly associated with warming climate (earlier ice-off), the changes are also consistent with data from higher trophic levels (fish health). By contrast, West Lake, with its sustained turbidity, continues to be dominated by lower-diversity benthic diatoms, additionally showing declining fish health.

Long-term climate records indicate warming across the Arctic since ca. 1850^3^. Threshold responses to permafrost change have had major impacts on landscape stability^2,24^, hydrology^6^, increased groundwater contribution to runoff^14,35^, and aquatic ecosystems^7^. This study demonstrates a further important dimension of climate-permafrost induced change on Arctic lakes; rapid sustained hydrochemical changes point to increased permafrost thaw and soil water drainage generating a downstream impact in large lakes on a timescale similar to what has been recorded in much smaller Arctic ponds^8^. This work indicates that permafrost thaw is an important mechanism for rapid alteration of Arctic freshwater systems that has been previously undemonstrated in continuous permafrost regions. These dynamics are important to characterize because the rapid shift in lake chemistry affects both Arctic water quality and aquatic ecosystems.

## Materials and Methods

Environmental monitoring has been carried out at CBAWO since 2003 as part of a long term watershed and terrestrial research program. Continuous meteorological observations and seasonal hydrological and limnological measurements represent a core data set, with manual sampling of water chemistry and lake water column profiling during summer periods during field activities. Soil (<1 m) and deeper (7.5 m) borehole temperature measurements began in 2011–12. Aquatic sampling was undertaken in 2003–4 and again in 2014 for diatoms and fish sampling was undertaken between 2008–15 for contaminant analysis.

Meteorological data was recorded at an automated meteorological station (WestMet, 80 m asl) from 2003–16. Temperature was recorded at WestMet with an Onset UA-003 temperature (0.1 °C accuracy) rainfall logger equipped with a Davis Industrial tipping bucket gauge (0.2 mm tip) positioned 1.5 m above the ground^9^.

Melt season hydrochemical data is initially available from 2003–04 for both lakes and then for 2008–16 and 2006–16 for the East and West Lakes, respectively. Water column electrical conductivity, temperature, turbidity and dissolved oxygen were measured with repeat vertical instrument (CTD) casts and fixed bottom moorings during the melt seasons and over-winter moorings in the West Lake in 2008–9 and during the 2011–15 period. Lake water samples were sampled and measured for major ion concentrations and metal concentrations were sampled July 2003 and July 2015 but not in intervening years. Lake water samples were collected in a 0.4 or 2 L Kemmerer water sampler from fixed stations located by GPS coordinates of each respective lake. Samples were recovered at depth intervals of 4–5 meters (year-dependent) to the lake bottom at approximately 32 m in the West and 30 m in the East Lake. Samples were collected in 1 L bottles that were rinsed three times with lake water before filling completely without headspace. Samples were processed within hours of collection and were vacuum filtered through a 0.22 µm polycarbonate membrane filter. The filtrate of each sample was collected in two 25 mL scintillation vials without headspace and was kept cool and dark in the field until returned to the laboratory for analysis. Water samples were shipped to Queen’s University where ion analysis was performed on a Dionex ICS 3000 ion chromatograph. Anions (Cl^−^, SO_4_^2−^, NO_3_^−^,) were separated by gradient elution with 16–40 mM potassium hydroxide, and cations (Na^+^, K^+^, Mg^2+^, Ca^2+^) were measured isocratically with 16 mM methanesulfonic acid eluent^9,25^. In 2003 and 2004, analysis was conducted at the Analytical Services Unit (ASU) at Queen’s University using chromatography for ions (Cl^−^, SO_4_^2−^, NO_3_^−^, Na^+^, K^+^, Mg^2+^, Ca^2+^). Metals were analyzed at ASU using ICP-MS (Al, Ba, Ca, Fe, Mg, Mn, Ni, K, Na, Sr, Ti, Zn) and ICP-OES (S)^36^.

Arctic char were sampled from 2008 to 2015 as part of a study investigating mercury cycling in freshwater ecosystems that have been impacted by permafrost. In 2013, a total of 46 fish were sampled (21 from West Lake, 25, from East Lake) and in 2015 a total of 30 fish were sampled (18 from East Lake, 10 from West Lake, and 2 from Headwater Lake). Physical characteristics of char including length and weight were recorded and otoliths were extracted for aging and chemical analysis. Ages of fish collected in 2013 and 2015 ranged from 12 to 27 years (East Lake) and 15 to 32 years in West Lake with a mean age of 18. Otoliths were removed on site and transported to Burlington, Ontario, where they were sectioned and aged with low power microscopy^37,38^. Fish relative condition, a measure of health using individual fish weight (W) and the predicted length–specific mean weight (W’)^39^, was calculated for all West and East lake fish from 2008–2016 with the exception of 2010. Sectioned otoliths were then transported to Queen’s Facility for Isotope Research (QFIR) in Kingston, Ontario, where they were analyzed by laser ablation inductively coupled plasma mass spectrometry (LA-ICPMS). A ThermoFinnigan Element 2 XR ICP-MS was couple to an Excimer 193 nm laser to perform chemical analysis. The laser spot size was 50 µm and ablated at a rate of 5 um/s. To create the maps, a series of 12–16 horizontal ablation lines were taken across the width of the otolith and stacked. External standards used include NIST 610, NIST 612, and MACS 3 (certified reference materials). Internal standard used was Ca.

Diatom sampling strategies for both 2004 and 2014 (this study) followed the same protocol^21^. All samples (total of 10 samples for each year) were collected from the same location at each lake at approximately one-week intervals during late June to early July in both sampling years. Littoral samples (all deposited into 20 ml scintillation vials) were retrieved from benthic habitats from a ~3 m^2^ submerged area: rock scrapes (each sample three rocks 10–20 cm in diameter brushed with a toothbrush) and littoral sediment (three samples of littoral sediment ~2 g each). To characterize water column habitats, mid-lake sediment trap samples^40^ were collected in 50-ml centrifuge tubes from ~0.5 m above the lake floor in the deepest part of the basin and retrieved on the same day as littoral samples.

All samples were preserved with several drops of Lugol’s solution. About 5 ml of each sample was processed for qualitative diatom assay encompassing overnight digestion in 15 ml sulphuric and nitric acids (50:50 molar ratio), followed by heating in a water bath (2 h at 90 °C) and subsequent repeated rinsing with distilled water until a neutral pH was obtained. Dilutions of residues dispersed onto coverslips and air-dried overnight were mounted in mounting medium (Naphrax or Zrax^21^). Slides were systematically scanned under light microscopy, counting a minimum of two transects (400–800 valves). Taxonomic identification follows Krammer & Lange-Bertalot^41,42^ and Antoniades *et al*.^43^.

## Electronic supplementary material

Supplementary figures
