# Supplementary material for: Climate and permafrost effects on the chemistry and ecosystems of High Arctic Lakes
Source: Sci Rep. 2017 Oct 16;7:13292. doi: 10.1038/s41598-017-13658-9 (PMC5643399; doi:10.1038/s41598-017-13658-9)
Supplement: Supplementary file 1 — Supplementary figures [file 41598_2017_13658_MOESM1_ESM.pdf]

Climate and permafrost effects on the chemistry and ecosystems of High Arctic Lakes

Authors:

K.E. Roberts[1], S.F. Lamoureux[1*], T.K. Kyser[2], D.C.G. Muir[3], M.J. Lafrenière[1], D. Iqaluk[4], A.J. Pieńkowski[5,6] and A. Normandeau[1,7]

[1]Department of Geography and Planning, Queen's University, Kingston, ON, K7L 3N6, Canada

[2]Department of Geological Sciences and Geological Engineering, Queen's University, Kingston, ON, K7L 3N6, Canada

[3]Environment and Climate Change Canada, Aquatic Contaminants Research Division, Burlington ON, Canada

[4]Resolute Bay, NU, Canada

[5]Department of Physical Sciences, MacEwan University, Edmonton, AB, T5J 4S2, Canada

[6] School of Ocean Sciences, College of Natural Sciences, Bangor University, Anglesey, LL59 5AB, UK

[7]Natural Resources Canada, Geological Survey of Canada Atlantic, Dartmouth, NS, B2Y 4A2, Canada

*Corresponding author: Scott F. Lamoureux, Department of Geography and Planning, Queen's University, Kingston, ON, K7L 3N6, Canada, scott.lamoureux@queensu.ca, Ph: 613-533-6030

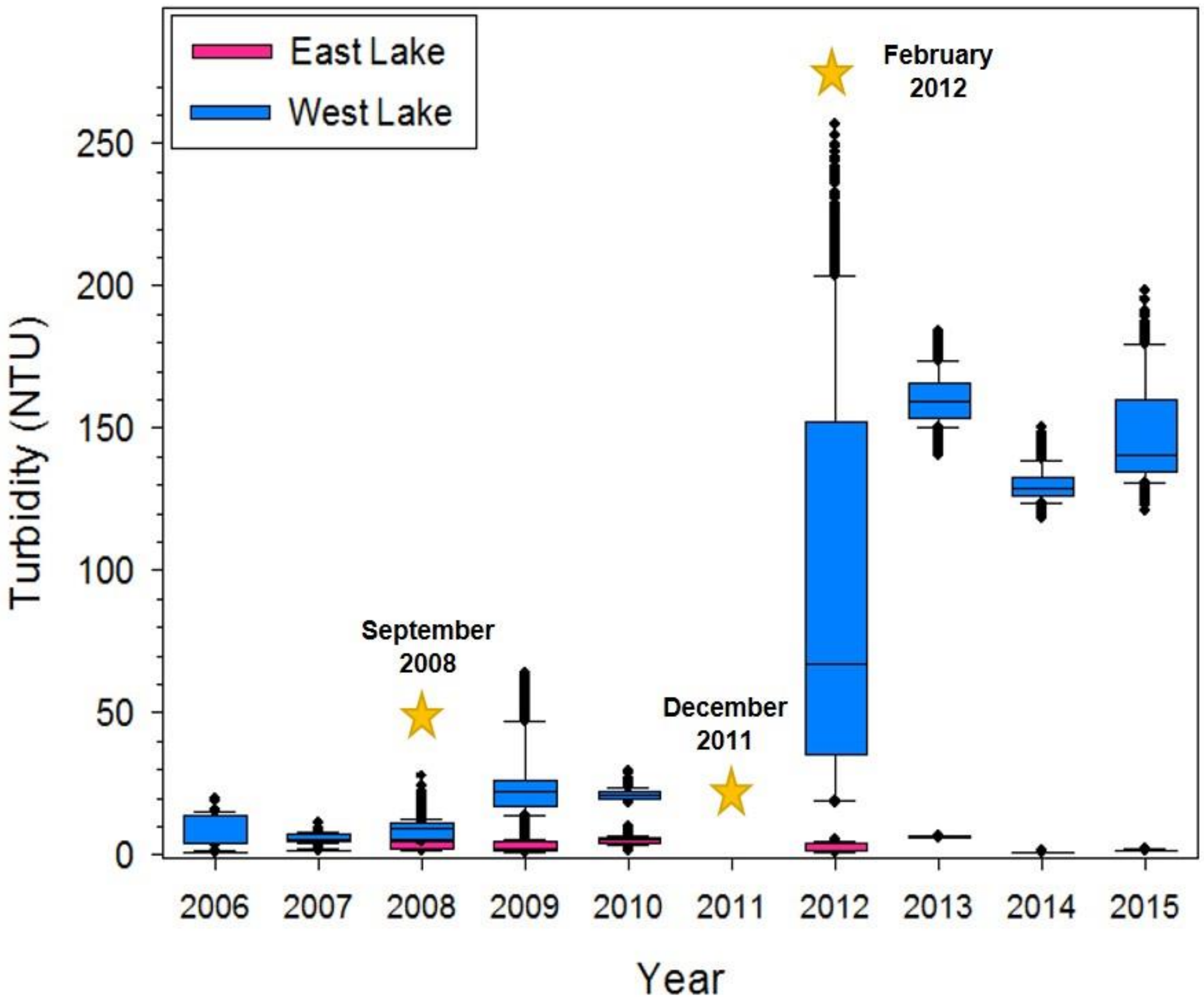

Supplementary Fig. S1: Turbidity (NTU) of East and West Lake (depths 10-20m) 2006-15. Stars indicated dates of subaqueous slumps observed in West Lake: September 2008, December 2011, and February 2012.

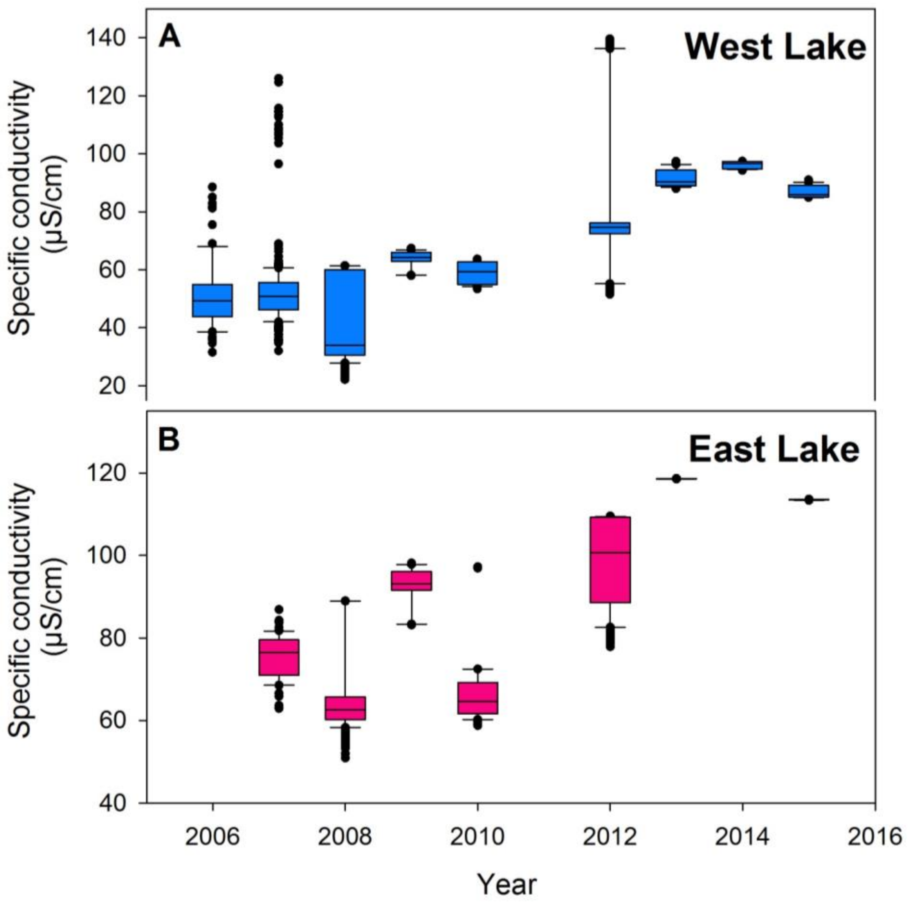

Supplementary Fig. S2: Specific conductivity (uS/cm) for A) West (2006-15) and B) East Lakes (2007-15) depths 10-20 m

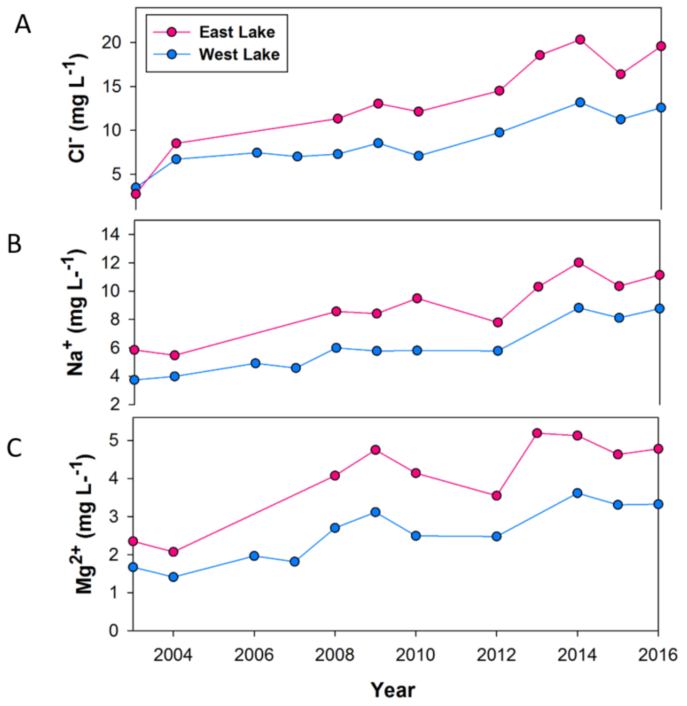

Supplementary Fig. S3: A) Cl^-, B) Na^+, and C) Mg^{2+} concentrations (mg L^{-1}) 2003-16 for East and West Lakes depths 10-20 m

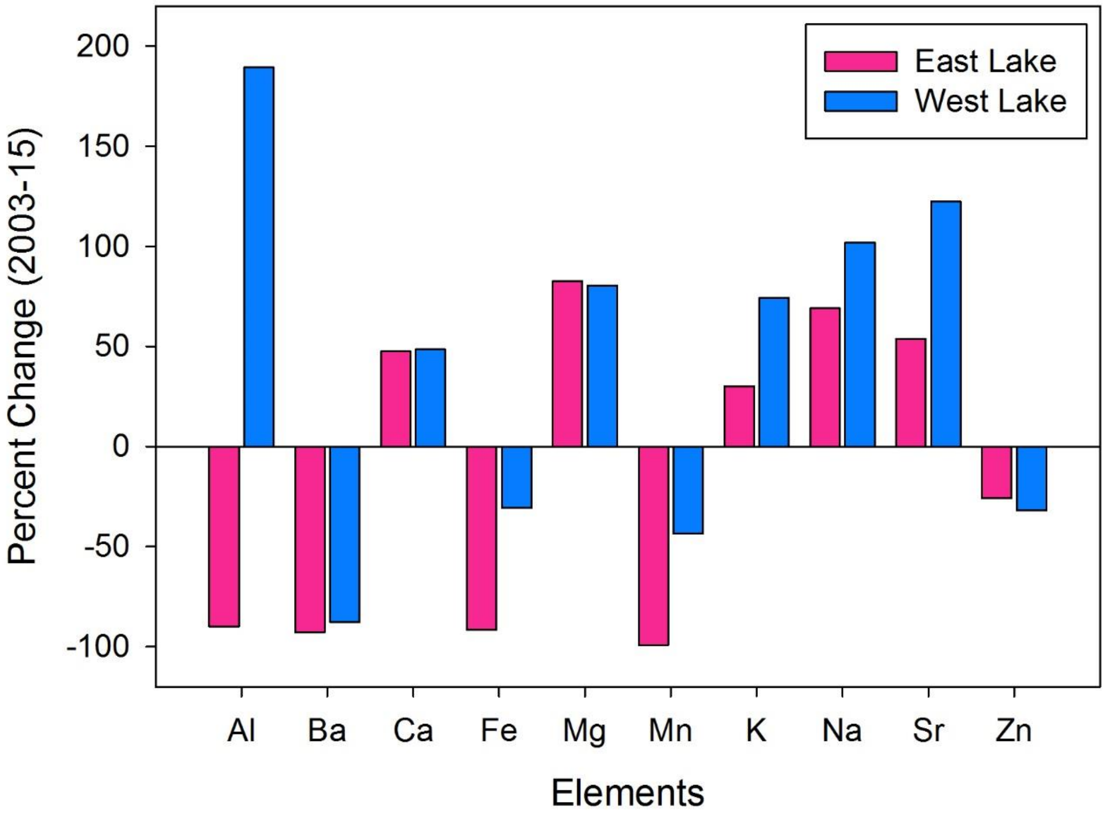

Supplementary Fig. S4: Percent change in metal concentrations (Al, Ba, Ca, Fe, Mg, Mn, K, Na, Sr, Zn) in East and West Lakes based on samples only from 2003 and 2015 (Other years not available).
